# Subjective well-being’s alterations as risk factors for major depressive disorder during the perimenopause onset: an analytical cross-sectional study amongst Mexican women residing in Guadalajara, Jalisco

**DOI:** 10.1186/s12905-022-01848-1

**Published:** 2022-07-05

**Authors:** Adrián Enrique Hernández-Muñoz, Ana Méndez-Magaña, Ana Lilia Fletes-Rayas, Miguel A. Rangel, Lenin Torres García, José de Jesús López-Jiménez

**Affiliations:** 1 Facultad de Medicina, Universidad Autónoma de Querétaro, Santiago de Querétaro, Mexico; 2 Universidad de Guadalajara, Guadalajara, Mexico; 3 CIBO, Centro de Investigación Biomédica de Occidente, Guadalajara, Mexico

**Keywords:** Perimenopause, Depression, Depressive disorder, major, Risk factor, Subjective well-being, Affective balance

## Abstract

**Background:**

Subjective well-being (SWB) can be defined as a self-report evaluation that reflects the satisfaction, and emotional level, over several social and personal indicators. Alterations in these indicators could become risk factors (RF) for major depressive disorder (MDD), but this association has not been studied at women’s life stages such as the perimenopause onset, despite its increasing prevalence for depressive symptomatology. Therefore, the aim of this study was to identify if SWB’s alterations determine RF for MDD during the perimenopause.

**Methods:**

An analytical cross-sectional study was realized in 252 Mexican women with perimenopause’s age range (48 ± 1.7) and menopausal symptomatology, treated on Medical Units belonging to Jalisco’s 13th Health-Region. We applied the INEGI’s *Basic Self-Reported Wellbeing Survey* (BIARE) that measured 30 SWB’s indicators. To identify MDD’s presence, the *Beck’s Depression Inventory-II* (BDI-II) was applied. The sample was studied with associative analysis, along with logistic regression models, to determine adjusted odds ratio (aOR) and corresponding 95% confidence interval (95% CI).

**Results:**

Trough the BDI-II we identified 40.5% women with MDD. When compared with the undepressed group we found lower scores in all the SWB’s indicators, along with significant associations for depressive symptomatology. However, the logistic regression allowed us to identify significant RF when the women specifically reported personal life-dissatisfaction (aOR 9.6, 95% CI 1.90–17.68), emotional imbalances between happiness/sadness (aOR 7.1, 95% CI 1.49–13.57) and concentration/boredom (aOR 6.7, 95% CI 1.43–13.48); free-time dissatisfaction (aOR 5.5, 95% CI 1.17–5.70), public security unconformity (aOR 5.4, 95% CI 2.20–11.3), and sense of purposelessness (aOR 4.2, 95% CI 1.07–19.41).

**Conclusion:**

The main objective of the study was to determine if SWB’s alterations are RF for depressive symptomatology, finding that social indicators with low scores are associated with MDD by means of aOR -Which were higher when compared to international research studies. Considering this, we suggest that more studies should be implemented, in order to understand and correctly attend the women’s social conditions during their perimenopause transition.

**Supplementary Information:**

The online version contains supplementary material available at 10.1186/s12905-022-01848-1.

## Background

The major depressive disorder (MDD) has become an important public health problem that represents the fifth-leading contributor to the Global Burden of Disease, and the fourth cause among the women [1]. Likewise, it has been reported that women’s life stages with hormonal changes become windows of vulnerability for the presence of MDD; especially the perimenopause stage that, when compared to premenopausal and postmenopausal stages, has a higher prevalence for altered mood states and somatic alterations -Along with an increasing likelihood for depressive symptomatology [2, 3].

To understand the causes of this affective disorder specifically during the menopause transition, many research papers have tried to analyze the women’s hormonal, clinical, and sociodemographic background [4]. However, as means to identify possible risk factors (RF) for MDD, recent studies have reported the importance of identifying alterations at the women’s social context [5, 6]. According to this, one of the variables that needs to be measured is the women’s Subjective Well-Being (SWB): a self-report measurement that reflects the personal experience in different elements [7], that measures the individual’s adaptive functioning for social conditions and societal progress [8].

Methodologically speaking, the SWB integrates multiple social indicators that can be divided into three main components: a cognitive component (CC), scoring the satisfaction level over different life domains; an affective component (AC), determining the emotional imbalance that a person have throughout the day; and an eudemonic component (EC), measuring the conformity degree between physical, emotional and adversity situations [7, 9]. The relationship between the alteration in these components and the prevalence for MDD’s symptomatology could be explained with the *Well-Being’s Homeostasis Theory*, which states that depression is a psychological state preceded by the loss of positive well-being [10].

Additionally, and considering the recommendations for the SWB’s periodical measurement stipulated by the *Organization for Economic Cooperation & Development* (OECD) and the *World Health Organization* (WHO) [11], the *Mexican Institute for Statistics & Geography* (INEGI) has identified more altered scores in women’s groups between 45 and 50 years old [12] -Same age range for the perimenopause onset in Mexican women (48 years ± 1.7) [13]. However, despite the latter, few research studies have explored if SWB’s lower scores could become associated RF for MDD, this is because most of the observational studies explore the linear correlation between the well-being’s indicators and the long-term development for depression [14].

The lack of these associations analyses, along with the increasing prevalence of depressive symptoms in Latin American women [15], makes the necessity to identify SWB’s alterations and their repercussions with the onset of MDD’s symptomatology. By understanding this associated interdependence, the results could promote future health care policies that pursue the correct attention of the women’s social conditions, especially during their menopause transition [16]. With this in view, the present study will seek to identify alterations in SWB’s indicators, and determine if they condition RF for the development of MDD amongst perimenopausal women.

## Methods

### Study design and ethical approval

To meet the objective, an analytical cross-sectional study was carried out through 2019’s Last Quarter and 2021’s First Quarter. This study was part of an extensive PhD thesis project, which sought to determine the clinical and psychosocial RF for MDD amongst Mexican women during their perimenopause stage, concluding in the summer of 2021.

Likewise, this project was evaluated, approved and consented by the Ethics Committee of the Jalisco’s 13th Health Region of the Central Zone of Guadalajara-Jalisco, which allowed the application of the research project in seven Medical Units that included medical, psychological and psychiatric care. The ethical approving code for the research project was 47/RXIII-JAL/2018.

Similarly, all the methods and data recollection were carried out in accordance with relevant guidelines and regulations, specifically the ethical principles of the Declaration of Helsinki. In accordance to this, we considered the ethical principles regarding the regulations of possible medical risks (16th to 22nd Principle), the guidelines concerning the research’s evaluation by a Research Ethical Committee (23rd Principle) and the recommendations to obtain a written-informed consent in all the patients (25th to 32nd Principles) [17].

### Sample characteristics

The sample was calculated by means of the statistical open source program OpenEpi 2.3, and was based on the 21.3% prevalence of depressive symptomatology amongst Mexican women residing in Jalisco [18]. Likewise, by considering a population of 10,854 users of public health services on the Jalisco Secretariat of Health -Medically treated throughout 2018 and 2019- and a 95% level of confidence, we obtained a sample of 252 women.

For the selection of the participants we developed a consecutive case series by inviting those women with the age range of interest (48 years ± 1.7) [13], along with the presence of menopausal symptomatology and amenorrhea in the last 12 months, which was previously checked in their medical records. We included those women who were waiting for clinical attention at the selected Health Centers, and who accepted to participate by signing a written informed consent letter [17]. We excluded those with a clinical background of artificial menopause, or neurocognitive disorders that could prevent them from participating in the research.

### Study questionnaires

Subsequently, we applied the *Beck's Depression Inventory II* (BDI-II) as a screening tool for the detection of MDD in the last two weeks [15]. With 83.3% sensitivity, 86.8% specificity and a 0.96 Cronbach’s alpha coefficient, the BDI-II uses a 0–3 Likert scale and a cutoff point of ≥ 14 scale to detect the presence and absence of MDD [19]. Alongside with the BDI-II, we applied the *Basic Self-Reported Well-Being Survey* (BIARE) to score SWB’s indicators, which was developed by the INEGI considering the OECD’s Guidelines [9], and has a Cronbach’s alpha coefficient of 0.91 [20].

The BIARE has a total of 30 SWB’s indicators measured by a 0–10 Likert scale, and distributed into four main sections: life satisfaction level, life domains, eudaimonic statements and emotional balance [12]. The first two sections set up the BIARE’s CC and, through the means of 14 indicators, measure the currently satisfaction level, along with the satisfaction over personal life aspects and public sphere domains (I.e. Public security, Country’s security satisfaction, and Residential area satisfaction) [9].

The third section of the BIARE scored the EC with the use of eleven statements: ten of them reflected the level of agreement or conformity with the person’s surroundings, and one negative statement described the adversity level and inability to return to a normal state [12]. Finally, the last section of the BIARE evaluated the AC’s imbalance throughout the day by employing five positive mood indicators, along with five negative ones that represented their counterpart which -When subtracted- resulted in five statements with scores that ranged from − 10 to 10.

### Variables measurement and statistical analysis

Normally, the BIARE’s indicators can be expressed as quantitative data, but depending on their score value they can also be grouped into qualitative categories. In the case of the 14 CC’s indicators and 11 EC’s statements, there are four categories: Very Dissatisfied/Strongly Disagree (Score values of 0 to 4), Dissatisfied/Disagree (5–6), Satisfied/Agree (7–8), and Very Satisfied/Strongly Agree (9–10). Meanwhile, there are three categories for the AC’s score: a negative group (Scores from − 10 to − 1), along with two positive score groups that range from 0 to 5 and from 6 to 10 [12].

However, to facilitate the bivariate and logistic analysis, two cutoff points were re-assigned for the CC and EC’s indicators: score values from 0 to 6 were used to identify SWB’s Alterations/Disagreements, and score values from 7 to 10 were used to determine Satisfaction/Conformity. Similarly, two value groups were established for the five AC’s statements: negative scores that ranged from − 10 to − 1, and positive scores that ranged from 0 to 10.

After employing the BDI-II we identified MDD symptomatology, which allowed the grouping of the sample into a depressed (≥ 14) and a non-depressed group (< 14); subsequently applying the BIARE to obtain SWB’s *average* (${\bar{\text{x}}}$) and *standard deviation values* (SD), comparing them by mean differences and *t* test. Then, according to the cutoff points that we previously established, we expressed quantitative scores into qualitative data and contingency tables. By means of a χ^2^ analysis, 95% confidence intervals (95% CI) and a ≤ 0.05 *p *value, we determined *odds ratio* (OR) and their statistical significance.

Finally, we included all the variables with a ≤ 0.25 *p* value into saturated logistic regression models, where we identified and eliminated all the confounding variables with a percent change analysis. This allowed us to identify *adjusted Odds Ratio* (aOR) for the determination of associated RF for MDD. All these analyses were made with the 22nd version of the Statistical Package IBM SPSS for Windows (IBM Corp., Armonk, New York, USA).

## Results

The application of the BDI-II allowed us to identify 102 women with depressive symptomatology (40.5% prevalence of MDD); dividing the sample into a depressed and a non-depressed group. Through the application of the BIARE survey, and by means of a *t* test, we found that the satisfaction levels among the CC’s indicators were lower on the depressed group (Table 1), especially in public sphere domains such as the personal satisfaction with their city (${\bar{\text{x}}}$ = 6.0 ± 2.8), country (${\bar{\text{x}}}$ = 5.8 ± 3.0), and public security (${\bar{\text{x}}}$ = 4.7 ± 3.3).

**Table 1:** CC* indicators scores regarding the presence of MDD**

Indicators	With MDD scores (${\bar{\text{x}}}$ ± SD)	Without MDD scores (${\bar{\text{x}}}$ ± SD)	Mean differences	*P* value
Life satisfaction				
Throughout the present year	7.1 ± 2.1	8.9 ± 1.1	1.8	0.001
Throughout the last year	6.8 ± 2.6	8.4 ± 1.7	1.6	0.001
Life domains				
Satisfaction with life	7.2 ± 2.2	8.8 ± 1.3	1.6	0.001
Satisfaction with the health level	6.9 ± 2.3	8.7 ± 1.3	1.8	0.001
Satisfaction with personal achievements	7.5 ± 2.5	8.9 ± 1.1	1.4	0.001
Satisfaction with personal relationships	7.5 ± 2.4	8.8 ± 1.6	1.3	0.001
Satisfaction with future perspectives	7.1 ± 2.6	8.8 ± 1.2	1.7	0.001
Satisfaction with the personal free time	6.0 ± 3.3	8.3 ± 2.0	2.3	0.001
Satisfaction with the public security	4.7 ± 3.3	7.1 ± 2.7	2.4	0.001
Satisfaction with the principal activity	7.1 ± 2.6	8.9 ± 1.3	1.8	0.001
Satisfaction with the living place	7.2 ± 2.7	8.6 ± 1.6	1.4	0.001
Satisfaction with their neighborhood	6.6 ± 2.9	8.1 ± 2.0	1.5	0.001
Satisfaction with the city of residence	6.0 ± 2.8	7.4 ± 2.3	1.4	0.001
Satisfaction with the country of residence	5.8 ± 3.0	7.0 ± 2.5	1.2	0.001

*Cognitive component, **major depressive disorder

${\bar{\text{x}}}$ = mean, SD = standard deviation

As for the EC’s statements (Table 2), the group of women with MDD presented low score values in all the positive valence indicators, being the statement with lowest ranking the one that scores the women’s feeling of fullness with themselves (${\bar{\text{x}}}$ = 6.9 ± 2.4). Moreover, when evaluating the adversity level statement, women from the depressed group showed higher scores within the inability to return to a normal state after feeling bad with themselves (${\bar{\text{x}}}$ = 7.0 ± 2.2).

**Table 2:** EC* statements scores regarding the presence of MDD**

Indicators	With MDD scores (${\bar{\text{x}}}$ ± SD)	Without MDD scores (${\bar{\text{x}}}$ ± SD)	Mean difference	*P* value
Eudaimonia/positive statements				
Feeling of fullness with oneself	6.9 ± 2.4	8.9 ± 1.2	2.0	0.001
Optimism with their own future	7.2 ± 2.7	8.9 ± 1.2	1.7	0.001
Freedom of choice in their own lives	7.4 ± 2.8	9.1 ± 1.6	1.7	0.001
Presence of strengths to adversities	7.6 ± 2.6	9.0 ± 1.4	1.4	0.001
Feeling that what they do is worth it	7.7 ± 2.5	9.4 ± 1.1	1.7	0.001
Feeling of fortune	7.4 ± 2.9	9.5 ± 1.0	2.1	0.001
Feeling that what happens is because of them	7.6 ± 2.6	9.0 ± 1.3	1.4	0.001
Feeling of purpose in their life	7.9 ± 2.5	9.3 ± 0.3	1.4	0.001
Importance of religion in their lives	7.4 ± 2.8	8.6 ± 2.1	1.2	0.001
Feeling of achievement throughout the day	7.4 ± 3.0	9.3 ± 1.0	1.9	0.001
Adversity/negative statements				
Inability to return to a normal state after feeling bad	7.0 ± 2.2	5.0 ± 3.1	-2.0	0.001

*Eudaimonic component, **major depressive disorder

${\bar{\text{x}}}$ = mean, SD = standard deviation

Subsequently, in relation to the AC’s imbalance (Table 3), the group of women diagnosed with MDD obtained lower scores in all the statements that measured the negative balance throughout their days, specifically the balance between good and bad humor (${\bar{\text{x}}}$ = 6.7 ± 2.5). Yet, the statements with higher scores were the ones that measured the concentration/boredom (${\bar{\text{x}}}$ = 7.6 ± 2.6) and happiness/sadness imbalance (${\bar{\text{x}}}$ = 7.7 ± 2.5).

**Table 3:** AC * imbalance statements scores regarding the presence of MDD **

Statements	With MDD scores (${\bar{\text{x}}}$ ± SD)	Without MDD scores (${\bar{\text{x}}}$ ± SD)	Mean differences	*P* value
Balance between good/bad humor	6.7 ± 2.5	8.8 ± 1.5	2.1	0.001
Balance between tranquility/concern	7.2 ± 2.7	8.9 ± 1.2	1.7	0.001
Balance between energy/fatigue	7.4 ± 2.8	9.1 ± 1.6	1.7	0.001
Balance between concentration/boredom	7.6 ± 2.6	9.0 ± 1.4	1.4	0.001
Balance between happiness/sadness	7.7 ± 2.5	9.4 ± 1.1	1.7	0.001

*Affective component, **major depressive disorder

${\bar{\text{x}}}$ = Mean, SD = standard deviation

Finally, we found significant associations within all the SWB’s indicators (Table 4). Nevertheless, the logistic analysis allowed us to determine RF for the MDD when the women reported personal life dissatisfaction (aOR 9.6, 95% CI 1.90–17.68), dissatisfaction with their free time (aOR 5.5., 95% CI 1.17–5.70), and with their public security (aOR 5.4, 95% CI 2.20–1.13). Similarly, the presence of emotional imbalances between happiness and sadness (aOR 7.1, 95% CI 1.49–13.57), concentration and boredom (aOR 6.7, 95% CI 1.43–13.48), and having no sense of purpose in life (aOR 4.2, 95% CI 1.07–19.41) were associated with the risk for depressive symptomatology.

**Table 4:** Bivariate and adjusted analysis of the BIARE* components to identify RF** for MDD***

Variables	With MDD		Without MDD		OR	95% CI^1^	*P* value	aOR ^2^	95% CI	*P* value
	No	%	No	%						
*Cognitive component*										
Life satisfaction throughout the present year										
With alterations (0–6)^1^	33	32.4	5	3.3	13.9	5.19–37.1	0.001	9.6	1.9–17.7	0.002
Without alterations (7–10)^2^	69	67.6	145	96.7	–	–	–	–	–	–
Life satisfaction throughout the last year										
With alterations (0–6)^1^	37	36.3	16	10.7	4.8	2.47–9.20	0.001	–	–	–
Without alterations (7–10)^2^	65	63.7	134	89.3	–	–	–	–	–	–
Satisfaction with life										
With alterations (0–6)^1^	32	31.4	6	4.0	11.0	4.38–27.5	0.001	–	–	–
Without alterations (7–10)^2^	70	65.7	141	94.0	–	–	–	–	–	–
Satisfaction with the health level										
With alterations (0–6)^1^	35	34.3	9	6.0	8.2	3.08–23.1	0.001	–	–	–
Without alterations (7–10)^2^	67	65.7	141	94.0	–	–	–	–	–	-
Satisfaction with personal achievements										
With alterations (0–6)^1^	23	22.6	5	3.3	8.4	3.08–23.1	0.001	–	–	–
Without alterations (7–10)^2^	79	77.4	145	96.7	–	–	–	–	–	–
Satisfaction with personal relationships										
With alterations (0–6)^1^	24	23.5	7	4.7	6.3	2.59–15.3	0.001	–	–	–
Without alterations (7–10)^2^	78	76.5	143	95.3	–	–	–	–	–	–
Satisfaction with future perspectives										
With alterations (0–6)^1^	30	29.4	6	4.0	10.0	3.98–25.1	0.001	–	–	–
Without alterations (7–10)^2^	72	70.6	144	96.0	–	–	–	–	–	–
Satisfaction with the personal free time										
With alterations (0–6)^1^	47	46.1	19	12.7	5.9	3.17–10.9	0.001	5.5	1.2–5.7	0.02
Without alterations (7–10)^2^	55	53.9	131	87.3	–	–	–	–	–	–
Satisfaction with the public security										
With alterations (0–6)^1^	68	66.7	48	32.0	4.3	2.49–7.26	0.001	5.4	2.2–11.3	0.02
Without alterations (7–10)^2^	34	33.3	102	68.0	–	–	–	–	–	–
Satisfaction with the principal activity										
With alterations (0–6)^1^	32	31.4	10	6.7	6.4	2.98–13.8	0.001	–	–	–
Without alterations (7–10)^2^	70	68.6	140	93.3	–	–	–	–	–	–
Satisfaction with the living place										
With alterations (0–6)^1^	25	24.5	15	10.0	2.9	1.45–5.9	0.001	–	–	–
Without alterations (7–10)^2^	77	75.5	135	90.0	–	–	–	–	–	–
Satisfaction with their Neighborhood										
With alterations (0–6)^1^	40	39.2	29	19.3	2.7	1.53–4.8	0.001	–	–	–
Without alterations (7–10)^2^	62	60.8	121	80.7	–	–	–	–	–	–
Satisfaction with the city of residence										
With alterations (0–6)^1^	46	45.1	38	25.3	2.4	1.42–4.1	0.001	–	–	–
Without alterations (7–10)^2^	56	54.9	112	74.7	–	–	–	–	–	–
Satisfaction with the country of residence										
With alterations (0–6)^1^	51	50.0	48	32.0	2.1	1.27–3.6	0.001	–	–	–
Without alterations (7–10)^2^	51	50.0	102	68.0	–	–	–	–	–	–
*Eudaimonic component*										
Feeling of fullness with oneself										
In disagreement (0–6)^1^	42	41.2	7	4.7	14.3	6.02–33.6	0.001	–	–	–
In agreement (7–10)^2^	60	58.8	143	95.3	–	–	–	–	–	–
Optimism with their own future										
In disagreement (0–6)^1^	34	33.3	7	4.7	10.2	4.31–24.2	0.001	–	–	–
In agreement (7–10)^2^	68	66.7	143	95.3	–	–	–	–	–	–
Freedom of choice in their own lives										
In disagreement (0–6)^1^	32	31.4	7	4.7	9.3	3.93–22.2	0.001	–	–	–
In agreement (7–10)^2^	70	68.6	143	95.3	–	–	–	–	–	–
Presence of strengths to adversities										
In disagreement (0–6)^1^	29	28.4	6	4.0	9.5	3.79–24.0	0.001	–	–	–
In agreement (7–10)^2^	73	71.6	144	96.0	–	–	–	–	–	–
Feeling that what they do is worth it										
In disagreement (0–6)^1^	28	27.5	4	2.7	13.8	4.67–40.9	0.001	–	–	–
In agreement (7–10)^2^	74	72.5	146	97.3	–	–	–	–	–	–
Feeling of fortune										
In disagreement (0–6)^1^	27	26.5	4	2.7	13.1	4.43–38.9	0.001	–	–	–
In agreement (7–10)^2^	75	73.5	146	97.3	–	–	–	–	–	–
Feeling that what happens is because of them										
In disagreement (0–6)^1^	27	26.5	10	6.7	5.0	2.32–11.0	0.001	–	–	–
In agreement (7–10)^2^	75	73.5	140	93.3	–	–	–	–	–	–
Feeling of purpose in their life										
In disagreement (0–6)^1^	27	26.5	3	2.0	17.6	5.18–60.0	0.001	4.2	1.1–19.4	0.04
In agreement (7–10)^2^	75	73.5	147	98.0	–	–	–	–	–	–
Importance of religion in their lives										
In disagreement (0–6)^1^	32	31.4	18	12.0	3.4	1.76–6.4	0.001	–	–	–
In agreement (7–10)^2^	70	68.6	132	88.0	–	–	–	–	–	–
Feeling of achievement throughout the day										
In disagreement (0–6)^1^	30	29.4	3	2.0	20.4	6.03–69.2	0.001	–	–	–
In agreement (7–10)^2^	72	70.6	147	98.0	–	–	–	–	–	–
Inability to return to a normal state after feeling bad										
In agreement (7–10)^3^	63	61.8	51	34.0	3.1	1.86–5.3	0.001	–	–	–
In disagreement (0–6)	39	28.2	99	66.0	–	–	–	–	–	–
*Affective component*										
Balance between good/bad humor										
Negative values (− 10 to − 1)	27	26.5	9	6.0	5.6	2.55–12.6	0.001	–	–	–
Positive values (0 to 10)	75	73.5	141	94	–	–	–	–	–	–
Balance between tranquility/concern										
Negative values (− 10 to − 1)	41	40.2	13	8.7	7.0	3.54–14.2	0.001	–	–	–
Positive values (0 to 10)	61	59.8	137	91.3	–	–	–	–	–	–
Balance between energy/fatigue										
Negative values (− 10 to − 1)	43	42.2	16	10.6	6.1	3.19–11.7	0.001	–	–	–
Positive values (0 to 10)	59	57.8	134	89.3	–	–	–	–	–	–
Balance between concentration/boredom										
Negative values (− 10 to − 1)	38	37.2	6	4.0	14.3	5.74–35.4	0.001	6.7	1.4–13.5	0.01
Positive values (0 to 10)	64	62.8	144	96.0	–	–	–	–	–	–
Balance between happiness/sadness										
Negative values (− 10 to − 1)	37	36.3	6	4.0	13.7	5.49–34.0	0.001	7.1	1.5–13.6	0.01
Positive values (0 to 10)	65	63.7	144	96.0	–	–	–	–	–	–

^*^Basic Self-Reported Well-Being Survey, **risk factors, ***major depressive disorder

^1^95% confidence intervals, ^2^Adjusted odds ratio

^1^Sum of the frequencies of “Very Unsatisfied” (0 to 4 scores) and “Unsatisfied” (5 to 6 scores) categories

^2^Sum of the frequencies of “Very Satisfied” (7 to 8 scores) and “Satisfied” (9 to 10 scores) categories

^3^In this variable the order of the rows was changed by taking into account the characteristic of the variable

## Discussion

The menopause transition is a natural process that involves physical, psychological, and social changes in the women; however, it has been reported that when this transition is negatively perceived, it conditions a decrease in the women’s emotional resilience [21]. The latter creates a cascading effect that affects the women’s SWB’s and subsequently causes the prevalence of depressive symptomatology [21, 22]. This last situation is reflected with the high prevalence of MDD that we found in our sample, which is similar to Taiwanese and Chinese research studies [23] but higher compared to other Mexican reports [24].

On the other hand, the INEGI studied women’s groups between 45 and 50 years old and residing on urban zones, which reported lower SWB’s scores in all of the CC’s indicators of satisfaction in contrast to other age groups. Comparing these results with the depressed group, we found a decrease of almost two units in the women’s satisfaction level, particularly with those belonging to the public sphere [25]. This dissatisfaction could be explained by the sociocultural perception that the Mexican population have with public aspects of their lives, such as their personal security and residential satisfaction, which can affect their daily activities and economic situation.

Similarly, the prevalence for adversity sensations with the AC’s statements of the depressed group was almost two times greater compared to the INEGI’s reports [24], possibly caused by an altered perception of the perimenopause transition. This is according to results from international cross-sectional and prospective studies, which have described that this rejection triggers long-term sensations of vulnerability and loss of control, modifying the women’s SWB degrees of conformity [26], their satisfaction levels, and in less quantity their emotional balance throughout the day [27].

Furthermore, the aOR of the CC and AC’s statements were four times greater than RF reported by British and Southeast England studies [6, 8]. This difference could be caused by the fact that the samples used in both researches involved women over 50 and 60 years old, which doesn’t present menopausal symptomatology or high SWB’s alterations -Thus presenting a lower presence for MDD symptomatology [28]. However, systematic reviews have explained that the menopause symptomatology doesn’t directly affect the women’s positive well-being [27]. Instead, the presence of MDD during this life period is directly influenced by an altered SWB, which it is related to the *Well-Being’s Homeostasis Theory* previously commented [10]

Subsequently, the aOR for the AC’s imbalance statements were two times greater than the RF reported in a North-American research. The reason for this difference could be explained by the fact that this last research measured the AC’s statements separately from the others SWB’s components. Regarding to this, it has been reported that most of the well-being’s scales -Applied during the perimenopause stage- focus more on the separate evaluation of its components and, unlike previous research studies [6], we used a SWB’s survey which measured and analyzed all of the main SWB’s indicators at once. This last situation, has led to the research proposal for the standardized use of SWB’s surveys that simultaneously measure all of its main indicators [29].

### Strengths, limitations and proposals

In view of the latter, the main strength of our research consists in the complete measurement of the main SWB’s components and indicators by means of the BIARE survey; analyzing the women’s social and personal context, by means of quantitative scores and qualitative data [12]. Throughout the understanding of these altered social indicators, and followed by the estimation of associated RF for MDD, our research explores a new etiological understanding for the depressive symptomatology at the menopause transition.

However, given the type of research design, one limitation in our research was the lack of analysis over previous chronical pathologies, economical factors and medical access, along with new SWB’s components such as the physical and perceived financial well-being which could influence the psychological health [29]. Similarly, this type of research design doesn’t allow follow-ups or a closer approach to the understanding of the psychosocial and geographical background that the Mexican women currently have with their surroundings.

Taking this into account, and given the geographical nature that some of the SWB’s indicators have, we suggest the future development of prospective research studies that allow clinical follow ups, as well as geocoding analysis [6, 9]. The last statement is related to what the OECD proposes with the public sphere domains, which can be used to understand the linear correlation between geographical context’s alterations and the presence of depressive symptomatology [9].

## Conclusion

Recently, the epidemiological exploration at the individual’s SWB has become an important objective of the WHO Health Policies [30]. This goal seeks the reduction of psychological disorders that affects a person’s life quality. So, by measuring the women’s SWB components during their perimenopause, and by comparing them with national and international reports, we expose the high prevalence of SWB’s alterations that the Mexican women have during their menopause transition, along with the risk that they entail for the presence of MDD.

Therefore in conclusion, we propose the development of future research projects that studies the women’s dissatisfaction levels with their social, personal or geographical context, especially in this stage of their lives. Considering the importance that the women’s SWB’s have in their emotional resilience, we suggest that the health personnel seek the correct measurement of social indicators -By means of scales that contemplate all the main components- throughout the clinical interviews. This last statement is expressed so that any social alterations can be identified, thus helping for the reduction of the increasing prevalence that the depressive symptomatology have during the perimenopause stage.

## Supplementary Information

**Additional file 1: Quantitative dataset**. Excel (.csv) file with the results obtained from the 252 patients of the study, which includes their score values (0-10) to each of the 30 BIARE’s indicators: fourteen CC indicators (Columns B to C; Y to AJ), eleven EC’s statements (Columns D to N), along with five positive and five negative mood indicators measuring the AC’s imbalance (Columns O to X). Likewise, the last column (Column AK) represents the BDI-II qualitative results, reflecting the presence (**1**) or absence (**0**) of MDD.

**Additional file 2: Qualitative dataset**. Excel (.csv) file with the 252 BIARE results, grouped into qualitative categories. For the 14 CC’s indicators (Columns B to C; T to AE) and 11 EC’s statements (Columns D to N), there are two category codes: **1** means score values from 0 to 6; and **0** the score values from 7 to 10. Meanwhile, by subtracting the positive mood indicators whith their counterparts (Columns O to X from **Additional File** 1) we classified the AC’s scores (Columns O to S) into two codes: **1** means score values from − 10 to – 1; **0** means results from 0 to 10. The last column reflects the presence (1) or absence (0) of MDD by means of the BDI-II.

## Data Availability

The datasets generated and analyzed during the current study are available in Additional file 1 [Quantitative Dataset].csv and Additional file 2 [Qualitative Dataset].csv.

## Abbreviations

MDD: Major depressive disorder
SWB: Subjective well-being
RF: Risk factors
CC: Cognitive component
EC: Eudemonic component
AC: Affective component
BDI-II: Beck Depression Inventory II
BIARE: Basic Self-Reported Well-Being Survey
INEGI: Mexican Institute of Statistics and Geography
OECD: Organization for Economic Cooperation and Development
WHO: World Health Organization
$${\bar{\text{x}}}$$: Average
SD: Standard deviation
OR: Odds ratio
aOR: Adjusted odds ratio
95% CI: 95% confidence intervals
